# Integrated 16S rRNA and Metagenomic Analysis of Pulmonary Microbiota in Sheep with Pneumonia

**DOI:** 10.3390/vetsci13070679

**Published:** 2026-07-13

**Authors:** Kehamo Abi, Zihan Xia, Lanmuyi Gou, Wentao Zhang, Kegu Ji’e, Shenglin Li, Taichun Gao, Wangqing Banma, Falong Yang

**Affiliations:** 1 Key Laboratory of Veterinary Medicine of Universities in Sichuan, Southwest Minzu University, Chengdu 610041, China; abikehamo@swun.edu.cn (K.A.); xiazh22@163.com (Z.X.); 15828471865@163.com (L.G.); 17882085261@163.com (W.Z.); 15718073975@163.com (K.J.); lsldwyzh66@126.com (S.L.); gseason1353@163.com (T.G.); 2 Animal Disease Prevention and Control Center of Aba Prefecture, Barkam 624000, China; 17345528071@163.com

**Keywords:** sheep, pneumonia, bacterial metagenomics, lung microbiome

## Abstract

Sheep are a major livestock species in China, yet pneumonia-related mortality poses a significant obstacle to intensive farming. In this study, 115 sheep lung samples were collected and classified into different pneumonia severity groups based on lung lesion scoring. Subsequently, this study employed 16S rRNA sequencing to systematically investigate the structure and diversity of the pulmonary microbiota in sheep, including alpha diversity, beta diversity, and LEfSe analyses. Metagenomic techniques were also applied to analyze the abundance of metabolic pathways, exploring the associations between functional gene differences and pneumonia severity, as well as putative antibiotic resistance genes, virulence factors, and the species contributions of functional genes in severe pneumonia cases. Microbial richness and diversity were significantly higher in the severe pneumonia group than in the healthy/mild lesion group (*p* < 0.05). While the dominant microbial structures were similar across the groups, notable differences were observed in the abundance of respiratory disease-associated genera, with *Pasteurella*, *Mannheimia*, *Mycoplasma*, *Bibersteinia*, and *Moraxella* identified as significantly enriched in severe cases. Moreover, several genera originating from the gut and oral cavity were also associated with pneumonia, suggesting a potential gut–lung axis. Carbohydrate metabolism was the most prevalent pathway in all groups, whereas amino acid metabolism was significantly enriched in the severe pneumonia group. Putative antibiotic resistance genes were differentially enriched; the severe pneumonia group showed significant enrichment of genes conferring resistance to aminoglycosides, tetracyclines, and polymyxins. Virulence factor analysis identified nutritional/metabolic factors and adhesion as the predominant virulence mechanisms. Species contribution analysis further revealed that *Mannheimia*, *Mycoplasma*, *Pasteurella*, and *Moraxella* were the predominant species associated with functional gene enrichment. In conclusion, the current study reveals associations between changes in the pulmonary microbiota structure and function and the severity of pneumonia in sheep, aiming to provide a foundation for future hypothesis-driven research on the role of the pulmonary microbiota in pneumonia progression.

## Simple Summary

Pneumonia is a serious and often fatal disease in sheep, causing substantial economic losses to farmers in China. To characterize the pulmonary microbiota across different severities of pneumonia, we analyzed lung samples from 115 sheep with varying degrees of pneumonia using 16S rRNA sequencing and metagenomics. Our results revealed that sheep with severe pneumonia harbored a significantly greater diversity of bacteria compared with those in the healthy/mild lesion group. Several genera associated with severe pneumonia, including the well-recognized respiratory pathogens *Pasteurella*, *Mannheimia*, *Mycoplasma*, *Bibersteinia*, and *Moraxella*, were markedly more abundant in severe cases. Interestingly, we also detected bacteria typically found in the gut or oral cavity, suggesting a potential association between the gut microbiome and pulmonary health. Furthermore, these bacteria carried genes associated with resistance to common antibiotics and the ability to attach to and invade lung tissue. By revealing how the lung microbial community composition is associated with pneumonia severity, this study provides a foundation for future hypothesis-driven research on the role of the pulmonary microbiota in pneumonia progression.

## 1. Introduction

Sheep are vital economic livestock globally, but respiratory diseases, particularly sheep pneumonia, are common in intensive farming, resulting in substantial economic losses [1,2,3]. The pathogens that cause sheep pneumonia include bacteria, respiratory viruses, mycoplasma, and parasites [1,2,4]. Research indicates that common pathogens causing pneumonia include *Pasteurella multocida*, *Mannheimia haemolytica*, *Klebsiella pneumoniae*, *Mycoplasma ovipneumoniae*, peste des petits ruminants virus, parainfluenza 3 virus, ovine adenovirus, and lungworms [5,6,7]. These pathogens frequently co-infect hosts, and their high variability complicates the diagnosis and treatment of sheep pneumonia [8].

Complex microbial communities and emerging pathogens pose challenges in identifying the pathogens responsible for sheep pneumonia. Metagenomics has been demonstrated to effectively identify a wide range of known and novel pathogens [9,10,11,12]. Unlike traditional diagnostic methods, metagenomic and metaviromic approaches can analyze all DNA or RNA present in samples, providing a comprehensive understanding of the composition and genetic characteristics of pathogenic microorganisms [13]. This systematic strategy enhances diagnostic precision and serves as a valuable tool for probing host–pathogen interactions and the underlying mechanisms of disease.

Lung microbiota significantly influences the onset and progression of pneumonia [12,14,15,16,17]. Research has shown that an imbalance in lung microbiota composition is closely linked to the development of lung diseases [18]. Virulence factors such as capsules and lipopolysaccharides enable lung pathogens to evade host immunity, damage lung tissue, promote infection, and trigger inflammatory responses [19,20]. However, the specific mechanisms remain unclear. Thus, investigating the key bacteria and viruses associated with unexplained sheep pneumonia will aid in accurate identification and prevention in intensive farming environments.

Here, we analyzed lung samples from 115 sheep with varying pneumonia severity, characterizing pulmonary microbial composition, virulence factor distribution, and their associations with disease severity. Our goal was to identify genera linked to severe pneumonia, providing insights that may guide future prevention and control efforts for this economically important disease.

## 2. Materials and Methods

### 2.1. Sample Collection

Lung samples were obtained from 115 commercial farm-sourced sheep (aged 4–5 months) originating from Qinghai, Xinjiang, Inner Mongolia, Gansu, and Ningxia. Among these, two animals were clinically healthy, while the remainder displayed respiratory symptoms. After an overnight fast, all animals were euthanized, and the thoracic cavity was opened to expose the lungs, which were then excised by severing the trachea. Bronchial mucus was collected from the left and right main bronchi using sterile cotton swabs pre-moistened with 0.01 M phosphate-buffered saline (pH 7.3). The swabs were immediately snap-frozen in liquid nitrogen, placed in 1.5 mL sterile microcentrifuge tubes, and stored at −80 °C until further processing.

### 2.2. Pulmonary Lesion Scoring and Grouping

Lung lobes were placed on aluminum foil and photographed under standardized lighting conditions. Pneumonia severity was evaluated by gross examination of lung photographs according to established criteria [21]. Each lung was divided into five lobes—right upper, right middle, right lower, left upper, and left lower—and a score from 0 to 5 was assigned to each lobe based on the percentage of the surface area affected: 0, no visible lesions; 1, <5%; 2, 5–25%; 3, 25–50%; 4, 50–75%; and 5, 75–100%. The scores from all five lobes were summed to generate a total lesion score, which was used to categorize pneumonia severity as follows: <12, healthy/mild; 12 to <24, moderate; and ≥24, severe. Given that only two animals were completely lesion-free (score = 0), they were combined with the mild lesion group (score 1–11) to form the healthy/mild group, as the sample size precluded meaningful separate statistical analysis.

### 2.3. 16S rRNA V3–V4 Region PCR Amplification, Library Construction, and Sequencing

Genomic DNA was extracted from 115 lung tissue samples using the OMEGA Soil DNA Kit (Omega Bio-tek, Norcross, GA, USA). DNA integrity and purity were evaluated via 0.8% agarose gel electrophoresis and spectrophotometry (NanoDrop NC2000, Thermo Fisher Scientific, Waltham, MA, USA). Following dilution to 20 ng/μL, the V3–V4 hypervariable region of the bacterial 16S rRNA gene was amplified with primers F (5′-ACTCCTACGGGAGGCAGCA-3′) and R (5′-GGACTACHVGGGTWTCTAAT-3′). Each 25 μL PCR mixture contained 5 μL of 5× reaction buffer, 5 μL of 5× GC buffer, 2 μL of dNTP mix (2.5 mM each), 1 μL of each primer (10 μM), 2 μL of template DNA, 8.75 μL of nuclease-free water, and 0.25 μL of Q5 DNA Polymerase. Thermal cycling was performed with an initial denaturation at 98 °C for 2 min, followed by 25–30 cycles of 98 °C for 15 s, 55 °C for 30 s, and 72 °C for 30 s, with a final extension at 72 °C for 5 min. Amplicons were resolved on 0.8% agarose gels, and target bands were excised and purified with the Axygen gel recovery kit (Axygen, Corning, NY, USA). Purified products were quantified with the Quant-iT PicoGreen dsDNA Assay Kit (Invitrogen, Carlsbad, CA, USA) using a microplate reader (BioTek, Winooski, VT, USA) and pooled in equimolar amounts. Sequencing libraries were constructed with the Illumina TruSeq Nano DNA LT Library Prep Kit (Illumina, San Diego, CA, USA) quality-controlled with the Agilent High Sensitivity DNA Kit (Agilent Technologies, Santa Clara, CA, USA), and quantified using the Quant-iT PicoGreen assay on a Promega QuantiFluor system (Promega, Madison, WI, USA). The finalized libraries were sequenced on the Illumina NovaSeq 6000 platform (Illumina, San Diego, CA, USA) with a 500-cycle SP Reagent Kit (Illumina, San Diego, CA, USA), generating 2 × 250 bp paired-end reads. All extraction and sequencing procedures were carried out by Shanghai Personalbio Biotechnology Co., Ltd. (Shanghai, China).

### 2.4. Sequencing Data Processing and Bioinformatics Analysis

After sequencing, the raw data were processed using the demux and cutadapt plugins for decoding and primer removal, followed by DADA2 for quality filtering and denoising. The sequences were merged at 100% similarity to generate Amplicon Sequence Variants (ASVs) and an abundance table. ASVs were compared against the Greengenes database for taxonomic information, and those with abundances below 0.001% of the total were removed. The processed data were uploaded to the Personalbio GeneCloud platform for analysis using QIIME2 (2019.4) and R (v3.2.0). QIIME2 performed random sampling of sequences at different depths to plot rarefaction curves, assessing if the sequencing depth was sufficient to reflect microbial diversity. The R “VennDiagram” package compared shared and unique ASVs between groups. Alpha and beta diversity analyses were conducted to explore variations in diversity and richness, and bar charts of species composition were created at various taxonomic levels. Finally, differential species were identified through LEfSe analysis and correlation heatmaps.

### 2.5. Metagenomic Library Construction and Sequencing

A total of 30 samples (10 per group) were selected from the healthy/mild lesion, moderate, and severe pneumonia groups for metagenomic sequencing. The selection was based on DNA quality (concentration ≥ 50 ng/μL, A260/A280 ≥ 1.8, assessed by Qubit 4 Fluorometer (Invitrogen, Carlsbad, CA, USA) and 1% agarose gel electrophoresis) and lesion scores representative of the median value of each severity group. Total DNA from all 30 lung microbiota samples was prepared using the Illumina TruSeq Nano DNA LT Library Prep Kit, following the Illumina TruSeq DNA Sample Preparation Guide. A metagenomic shotgun sequencing library with an insert size of 400 bp was constructed according to standard laboratory protocols.

### 2.6. Sequence Data Processing and Statistical Analyses

Quality filtering of raw reads was performed by removing adapter sequences with Cutadapt (v1.2.1) and trimming low-quality bases via a sliding-window approach. Host-derived reads were eliminated by alignment to the sheep reference genome using minimap2 (2.24-r1122). Taxonomic assignment of metagenomic reads was carried out with Kraken2 against a RefSeq-derived database, and reads classified as metazoan or viridiplantae were excluded. Assembly of clean reads for each sample was conducted with Megahit (v1.1.2) using the meta-large preset. Resulting contigs (>300 bp) were clustered with mmseqs2 in “easy-linclust” mode at 95% sequence identity and 90% coverage relative to the shorter contig. Taxonomic lineage of the non-redundant contigs was determined by alignment to the NCBI-nt database using mmseqs2 in “taxonomy” mode, with contigs assigned to Viridiplantae or Metazoa discarded. Gene prediction was performed with MetaGeneMark, and CDS sequences were clustered at 90% protein identity and 90% coverage using mmseqs2 “easy-cluster” mode. Abundance estimation was carried out by mapping quality-filtered reads to the predicted genes with Salmon in quasi-mapping mode (--meta --minScoreFraction = 0.55), and expression levels were normalized as CPM (copies per kilobase per million mapped reads). Functional annotation of the non-redundant genes was performed against KEGG, CARD, and VFDB protein databases using mmseqs2 in “search” mode with a sensitivity parameter of 5.7. For intergroup comparisons of functional gene abundances, raw *p*-values were applied as the significance threshold (*p* < 0.05). Downstream bioinformatics analyses, including rarefaction curve generation, species composition profiling, functional analysis, and species–function correlation assessment, were conducted on the Personalbio GeneCloud platform (https://www.genescloud.cn/home (accessed on 20 October 2025)).

### 2.7. Statistical Analysis

Statistical analyses were conducted using SPSS 18.0, and graphical representations were generated with GraphPad Prism 7.0 and the Personalbio GeneCloud platform (https://www.genescloud.cn (accessed on 6 November 2025)). Alpha diversity analysis was performed on the three sample groups using the Kruskal–Wallis rank-sum test and Dunn’s test, visualized as box plots. For beta diversity analysis, R and QIIME2 software were used based on the Bray–Curtis distance algorithm. Differences in species, resistant gene families, and virulence types at the taxonomic level among different pneumonia groups were analyzed and represented as box plots. Differences in relative abundance at various taxonomic levels were analyzed using the Kruskal–Wallis non-parametric test and Dunn’s test. Spearman’s rank correlation coefficients were calculated to evaluate relationships between variables, and the resulting correlation matrix was visualized as a heatmap using the psych and pheatmap R packages. For non-normally distributed datasets, intergroup comparisons were performed with the non-parametric Wilcoxon rank-sum test. Before using the Student’s *t*-test, normality (Shapiro–Wilk test) and homogeneity of variance (Levene’s test) were verified; when these assumptions were not met, the non-parametric Wilcoxon rank-sum test was used instead. In this study, a *p*-value of less than 0.05 was considered to indicate statistical significance.

## 3. Results

### 3.1. Lung Lesion Scoring and Grouping Results

**Figure 1:**
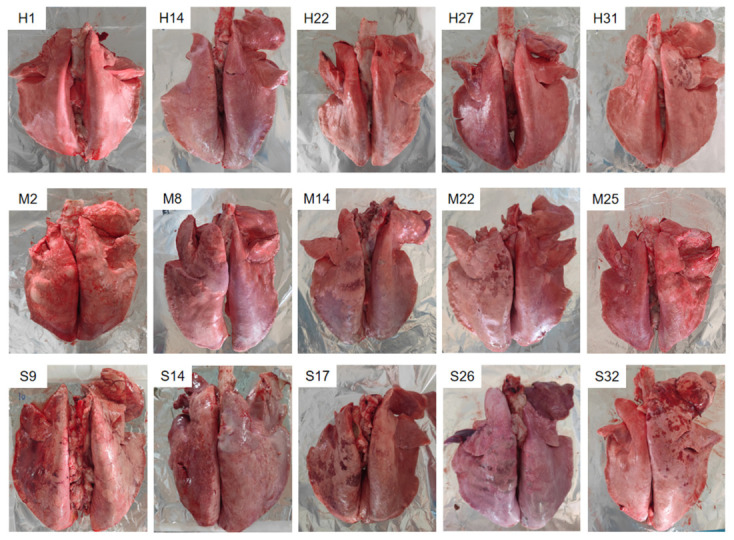
Representative lung images and lesion scores of different pneumonia severity groups. (H) healthy/mild lesion group; (M) moderate lesion group; (S) severe lesion group.

According to the lung lesion scoring criteria, the 115 lung samples collected for this study were scored and categorized into three groups (Figure 1): a healthy/mild lesion group (H) with 37 samples, a moderate lesion group (M) with 38 samples, and a severe lesion group (S) with 40 samples. The lesion scores ranged from 0 to 35, with mean scores of 5, 21, and 32 for the H, M, and S groups, respectively. It should be noted that the H group comprised animals with no visible lesions (score = 0, *n* = 2) and those with mild lesions (score 1–11), and is therefore not a true healthy control group.

### 3.2. 16S rRNA and Metagenomic Sequencing Quality Control Analysis

To determine whether the extracted DNA was suitable for subsequent library construction and sequencing, 0.8% agarose gel electrophoresis and NanoDrop NC2000 spectrophotometry were performed on 115 lung microbial total DNA samples. The results showed that the genomic DNA was not severely degraded, with clear bands observed around 2000 bp (Supplementary Figure S1), indicating good DNA integrity. All samples had concentrations exceeding 0.5 ng/μL, meeting the standards for library construction and sequencing. To verify if the data volume sufficiently covered most microbial species, rarefaction analysis was conducted based on the observed_species index, resulting in a rarefaction curve (Supplementary Figure S2). The results demonstrated that the curve reached a plateau with increasing sequencing depth, suggesting that the identified lung microbial taxa had reached saturation. Thus, the sequencing data generated in this work are sufficient for downstream analyses.

### 3.3. Diversity Analysis of Lung Microbial Communities in Varying Degrees of Pneumonia

**Figure 2:**
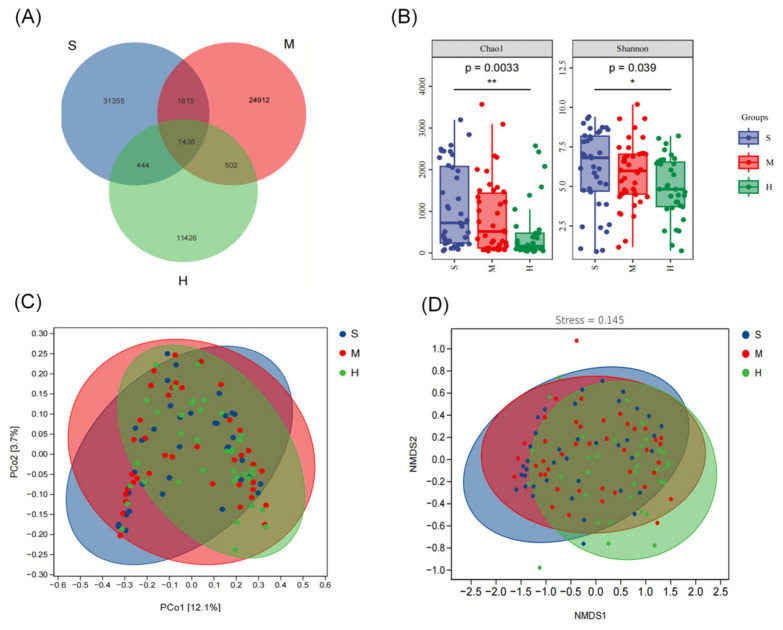
Analysis of lung microbiota diversity in sheep with pneumonia of varying severity. (**A**) ASV Venn diagram. (**B**) Boxplots of alpha diversity indices. (**C**) Principal coordinate analysis (PCoA) plot. (**D**) Non-metric multidimensional scaling (NMDS) plot. (* *p* < 0.05, ** *p* < 0.01).

To understand the richness and diversity of lung microbial communities across different groups, this study examined lung microbial richness and diversity across pneumonia groups using Venn diagrams and alpha diversity analyses. The Venn diagram (Figure 2A) revealed that the healthy/mild, moderate, and severe pneumonia groups shared 1436 ASVs, with unique ASVs numbering 11,426 for the healthy/mild and 31,355 for the severe pneumonia group, indicating greater richness in the severe group. Alpha diversity, assessed by Chao1 and Shannon indices (Figure 2B), revealed significantly greater richness (*p* < 0.01) and diversity (*p* < 0.05) in the severe group than in the healthy/mild group, whereas no significant difference was observed between the moderate and severe groups. Beta diversity analysis using PCoA (Figure 2C) and NMDS (Figure 2D) demonstrated strong clustering within each group, with limited separation between the severe and moderate groups. Notably, the microbial community structure of the healthy/mild group was clearly distinct from that of the severe pneumonia group.

### 3.4. Microbial Community Composition in the Lungs at Varying Degrees of Pneumonia

**Figure 3:**
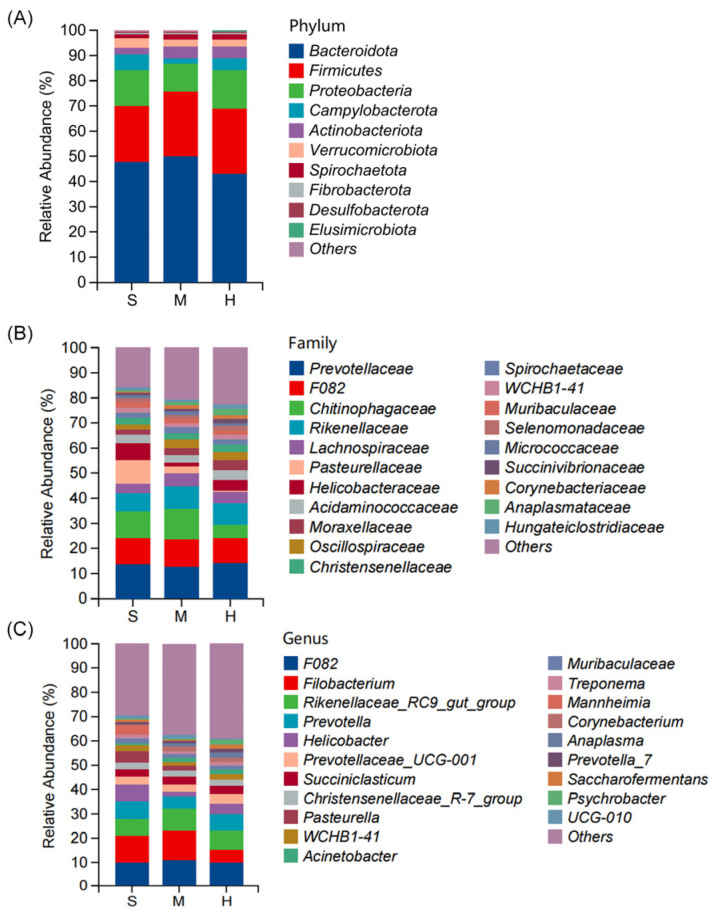
Taxonomic composition of the lung microbiota at the phylum, family, and genus levels. (**A**) Top 10 bar chart at the phylum level. (**B**) Top 20 bar chart at the family level. (**C**) Top 20 bar chart at the genus level.

To understand the structural characteristics of lung microbial communities at different taxonomic levels, this study utilized QIME2 software to conduct ASV species clustering analysis on sheep lung samples with varying degrees of pneumonia. A total of 27 phyla, 268 bacterial families, and 737 bacterial genera were identified across 115 lung samples. At the phylum level, the core phyla with an average relative abundance exceeding 1.00% in the healthy/mild, moderate, and severe pneumonia groups included 7 phyla (Figure 3A): *Bacteroidota* (43.4%, 50.0%, 47.7%), *Firmicutes* (25.5%, 26.0%, 22.1%), *Proteobacteria* (15.4%, 10.9%, 14.3%), *Campylobacterota* (4.4%, 2.1%, 6.6%), *Actinobacteria* (4.9%, 4.9%, 2.3%), *Verrucomicrobiota* (2.5%, 2.1%, 3.7%), and *Spirochaetota* (2.3%, 2.5%, 2.0%). At the family level, the core families with an average relative abundance exceeding 1.00% in the healthy/mild, moderate, and severe pneumonia groups included 22 families (Figure 3B): *Prevotellaceae* (14.1%, 12.7%, 13.8%), F082 (10.0%, 11.3%, 10.2%), *Chitinophagaceae* (5.2%, 12.0%, 11.0%), *Rikenellaceae* (8.6%, 9.0%, 7.1%), *Lachnospiraceae* (4.8%, 6.0%, 3.7%), *Pasteurellaceae* (0.45%, 2.5%, 9.5%), *Helicobacteraceae* (4.3%, 1.6%, 6.5%), *Acidaminococcaceae* (3.3%, 3.2%, 3.4%), *Moraxellaceae*, *Oscillospiraceae* (3.2%, 3.4%, 2.1%), *Christensenellaceae* (2.8%, 2.6%, 2.7%), *Spirochaetaceae* (2.3%, 2.5%, 2.0%), WCHB1-41 (1.7%, 1.5%, 2.5%), *Muribaculaceae* (1.4%, 1.3%, 2.0%), *Selenomonadaceae* (1.9%, 1.6%, 1.3%), *Micrococcaceae* (1.0%, 1.9%, 1.2%), *Succinivibrionaceae* (1.9%, 0.9%, 1.0%), *Corynebacteriaceae* (1.6%, 1.7%, 0.5%), *Anaplasmataceae* (2.4%, 1.0%, 0.2%), *Hungateiclostridiaceae* (1.4%, 0.7%, 1.3%), *Ruminococcaceae* (0.7%, 1.7%, 1.0%), and UCG-010 (0.8%, 1.1%, 1.1%). At the genus level, the core genera with an average relative abundance exceeding 1.00% in the healthy/mild, moderate, and severe pneumonia groups included 20 genera (Figure 3C): F082 (10.0%, 11.3%, 10.2%), *Filobacterium* (5.2%, 12.0%, 11.0%), *Rikenellaceae* RC9 *gutgroup* (8.1%, 8.6%, 6.9%), *Prevotella* (6.7%, 5.4%, 7.4%), *Helicobacter* (4.3%, 1.6%, 6.5%), *Prevotellaceae*_UCG-001 (4.0%, 3.5%, 3.2%), *Succiniclasticum* (3.2%, 3.0%, 3.4%), *Christensenellaceae*_R-7_group (2.8%, 2.6%, 2.7%), *Pasteurella* (1.1%, 2.1%, 4.7%), WCHB1-41 (1.7%, 1.5%, 2.5%), *Acinetobacter* (2.6%, 1.6%, 0.6%), *Muribaculaceae* (1.4%, 1.3%, 2.0%), *Treponema* (1.3%, 1.3%, 1.5%), *Mannheimia* (0.04%, 0.08%, 3.7%), *Corynebacterium* (1.6%, 1.7%, 0.5%), *Anaplasma* (2.4%, 1.0%, 0.2%), *Prevotella*_7 (1.6%, 1.2%, 0.7%), *Saccharofermentans* (1.4%, 0.7%, 1.3%), *Psychrobacter* (1.9%, 0.7%, 0.6%), and UCG-010 (0.8%, 1.2%, 1.1%). Additionally, analysis of the lung microbial composition at different taxonomic levels indicated that the dominant microbial structures in the severe and moderate pneumonia groups were similar to those in the healthy/mild pneumonia group, although there were differences in relative abundances. Furthermore, among the dominant communities at each taxonomic level, intestinal and plant-associated microbial groups were also identified, in addition to respiratory and oral microbiota.

**Figure 4:**
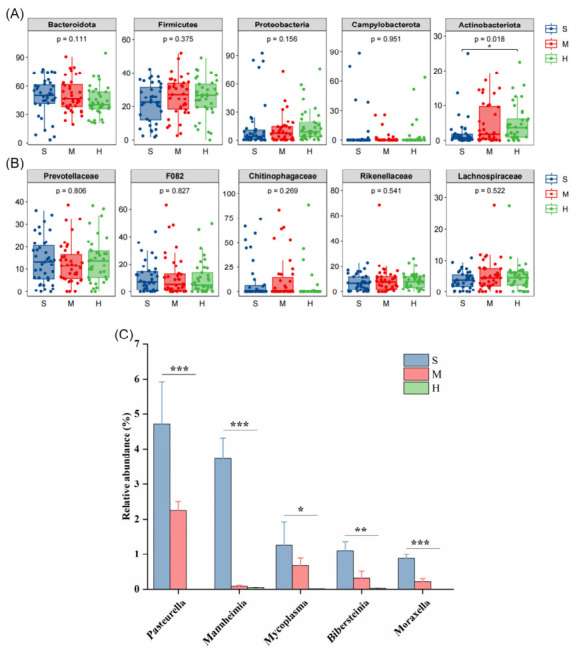
Differential abundance analysis of lung microbiota at the phylum, family, and genus levels. (**A**) Boxplots of the top 5 phyla with significant differences. (**B**) Boxplots of the top 5 families with significant differences. (**C**) Bar chart showing genera significantly enriched in severe pneumonia cases (* *p* < 0.05, ** *p* < 0.01, *** *p* < 0.001).

To understand the variations in lung microbial abundance and their correlation with pneumonia severity, this study analyzed dominant microbial communities at different taxonomic levels. The results showed that at the phylum level, *Bacteroidota* had a higher relative abundance in the moderate and severe pneumonia groups compared to the healthy/mild group, while *Firmicutes* was more abundant in the healthy/mild group than in the severe group (Figure 4A). At the family level, *Prevotellaceae* and F082 had higher relative abundances in the severe pneumonia group compared to the healthy/mild group, but these differences were not statistically significant (Figure 4B). At the genus level, the analysis focused on pathogenic bacteria associated with respiratory diseases. The results revealed that the relative abundances of *Pasteurella*, *Mannheimia*, *Mycoplasma*, *Bibersteinia*, and *Moraxella* were significantly higher in the severe pneumonia group than in the healthy/mild group (*p* < 0.05) (Figure 4C). These findings show that the severe pneumonia group had a greater abundance and diversity of respiratory-associated genera.

### 3.5. Identification of Genera Associated with Pneumonia Severity in Sheep

**Figure 5:**
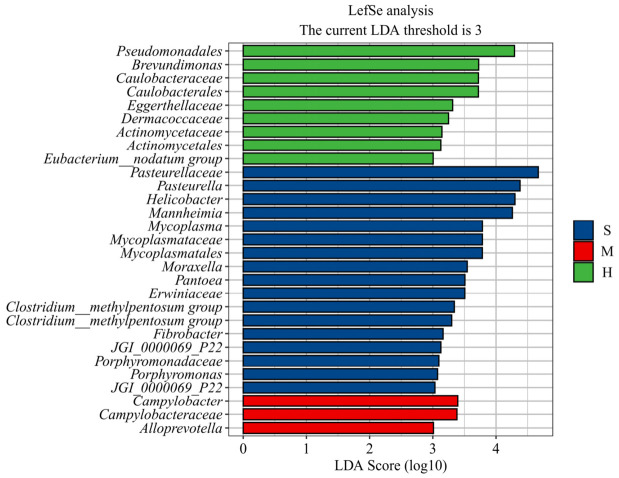
LEfSe analysis identifying differentially abundant taxa among groups. Bars represent linear discriminant analysis (LDA) scores (LDA > 3).

**Figure 6:**
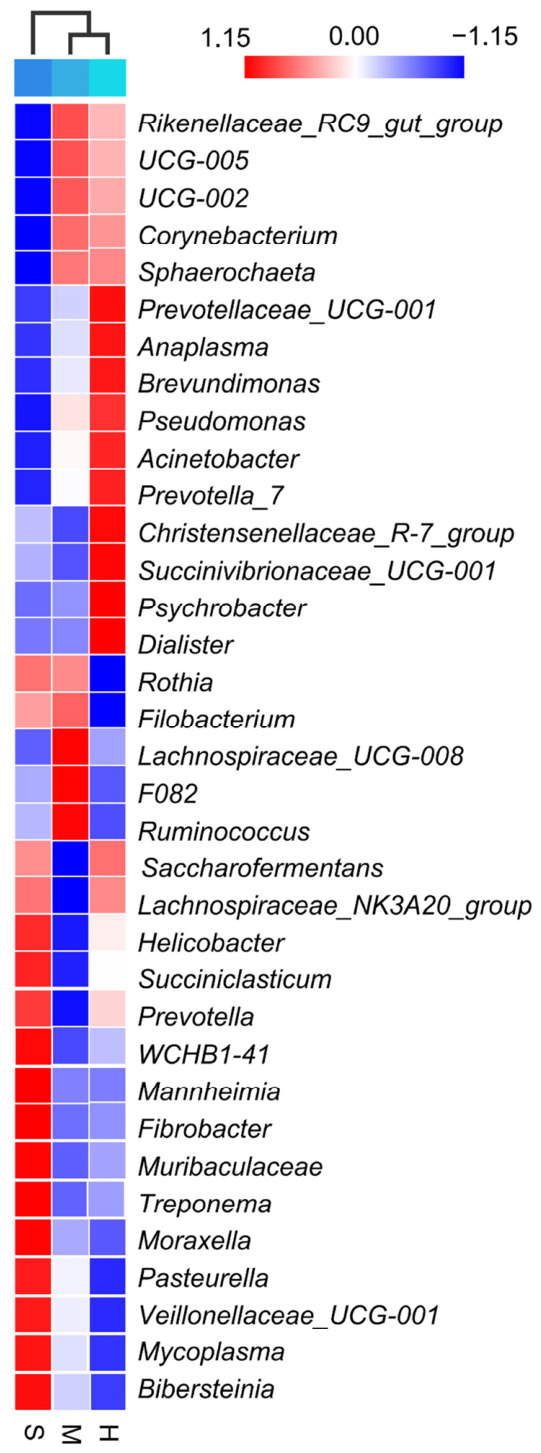
Correlation heatmap between bacterial genera and pneumonia severity at the genus level. (Red indicates positive correlation; blue indicates negative correlation; white indicates no significant correlation).

To identify microbial taxa associated with pneumonia severity in sheep, this study compared lung microbial communities across varying degrees of pneumonia using LEfSe analysis and correlation heatmaps. LEfSe was applied to identify biomarkers among the groups, integrating non-parametric Kruskal–Wallis and Wilcoxon rank-sum tests with linear discriminant analysis (LDA) for effect size estimation (Figure 5). The analysis revealed that *Alloprevotella* and *Campylobacter* were significantly enriched in the moderate pneumonia group (LDA > 3), while *Pasteurella*, *Mannheimia*, and *Mycoplasma*, associated with respiratory diseases in sheep, were significantly more abundant in the severe pneumonia group, identifying them as stable differential taxa (LDA > 3.5). Additionally, *Moraxella*, along with genera typically found in the gut, *Helicobacter* and *Pantoea*, showed significant abundance in the severe pneumonia group (LDA > 3.5). The correlation heatmap of species abundance and pneumonia severity indicated that, in addition to the genera enriched in the severe pneumonia group identified through LEfSe analysis, *Bibersteinia*, *Veillonellaceae*_UCG-001, *Treponema*, and *Prevotella* also exhibited strong correlations with severe pneumonia (Figure 6). This suggests that, besides respiratory pathogens, certain gut and oral microbiota may also be associated with pneumonia severity in sheep.

### 3.6. Differences in Lung Microbiome at Taxonomic Levels Among Different Degrees of Pneumonia

**Figure 7:**
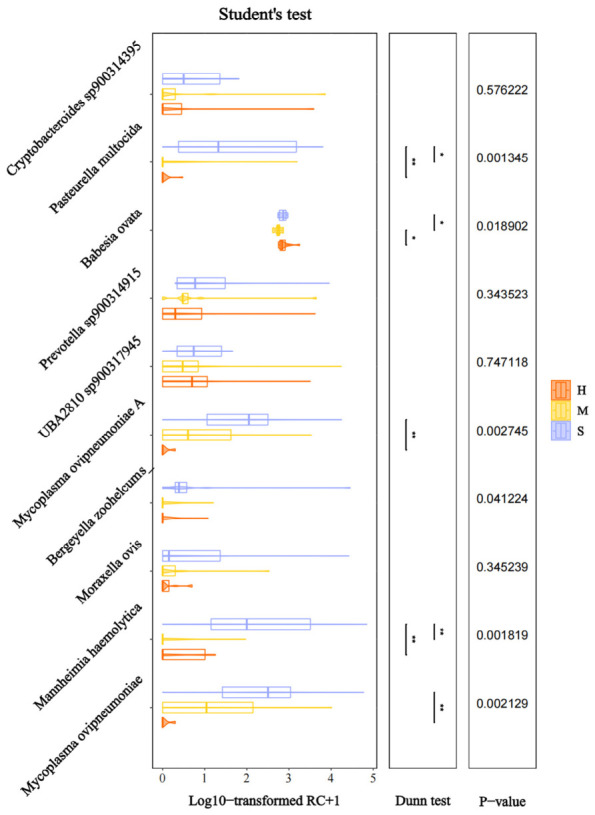
Differential abundance analysis of bacterial species across pneumonia severity groups. Only species with significant differences are shown (* *p* < 0.05, ** *p* < 0.01).

To further characterize the differences in the lung microbiome among varying degrees of pneumonia, this study conducted a Student’s t-test analysis on each pneumonia group. The results showed that the abundances of *Pasteurella multocida*, *Mycoplasma ovipneumoniae*, and *Mannheimia haemolytica* in the severe pneumonia group were significantly higher than those in the healthy/mild lesion group (*p* < 0.01). These three species are well-recognized respiratory pathogens and were significantly enriched in severe cases, suggesting a potential association with pneumonia severity. Additionally, in the severe pneumonia group, the abundances of *Moraxella ovis*, *Bergeyella zoohelcum*, *Cryptobacteroides* sp900314395, and *Prevotella* sp900314915 were higher compared to the healthy/mild lesion group, although the differences were not statistically significant (Figure 7).

### 3.7. Functional Gene Differences in the Lung Microbiome Across Pneumonia Severity Groups

**Figure 8:**
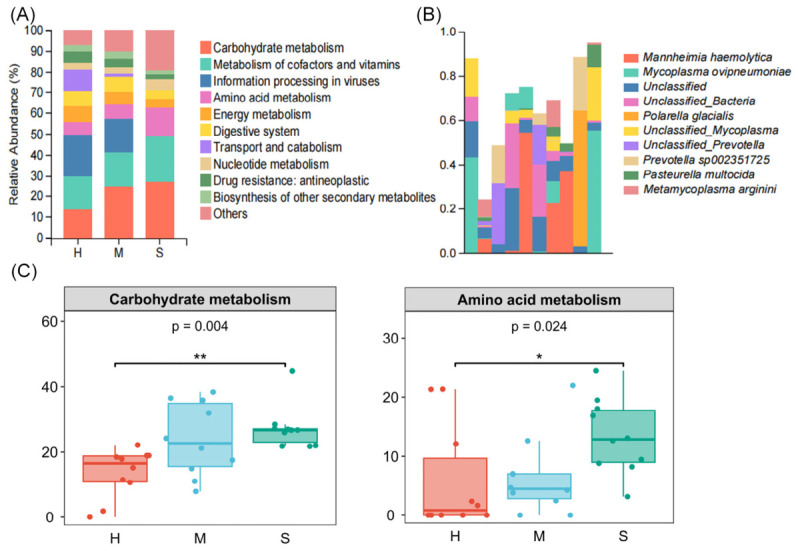
Metabolic pathway profiles of the lung microbiome across pneumonia severity groups. (**A**) Predicted functional pathway enrichment analysis. (**B**) Species contribution to carbohydrate metabolism in the severe pneumonia group. (**C**) Boxplots of significantly enriched metabolic pathways in the severe pneumonia group: carbohydrate metabolism (left panel) and amino acid metabolism (right panel) (* *p* < 0.05, ** *p* < 0.01).

To further examine the distribution of functional genes in the sheep lung microbiome across different pneumonia severities, this study employed metagenomic techniques to assess variations in metabolic pathways, putative antibiotic resistance genes, virulence factor abundances, and species contributions. The results showed that carbohydrate metabolism was the most abundant metabolic pathway across all pneumonia groups, with significant enrichment in the moderate and severe pneumonia groups (*p* < 0.01) (Figure 8A). This pathway primarily includes amino sugar and nucleotide sugar metabolism, glycolysis, and the pentose phosphate pathway. Species contribution analysis indicated that *Pasteurella multocida*, *Mycoplasma ovipneumoniae*, and *Prevotella* species were the predominant contributors to the carbohydrate metabolism pathway of the severe pneumonia group (Figure 8B). Additionally, the amino acid metabolism pathway was significantly enriched in the severe pneumonia group (*p* < 0.05) (Figure 8C), including biosynthetic pathways for lysine and phenylalanine.

**Figure 9:**
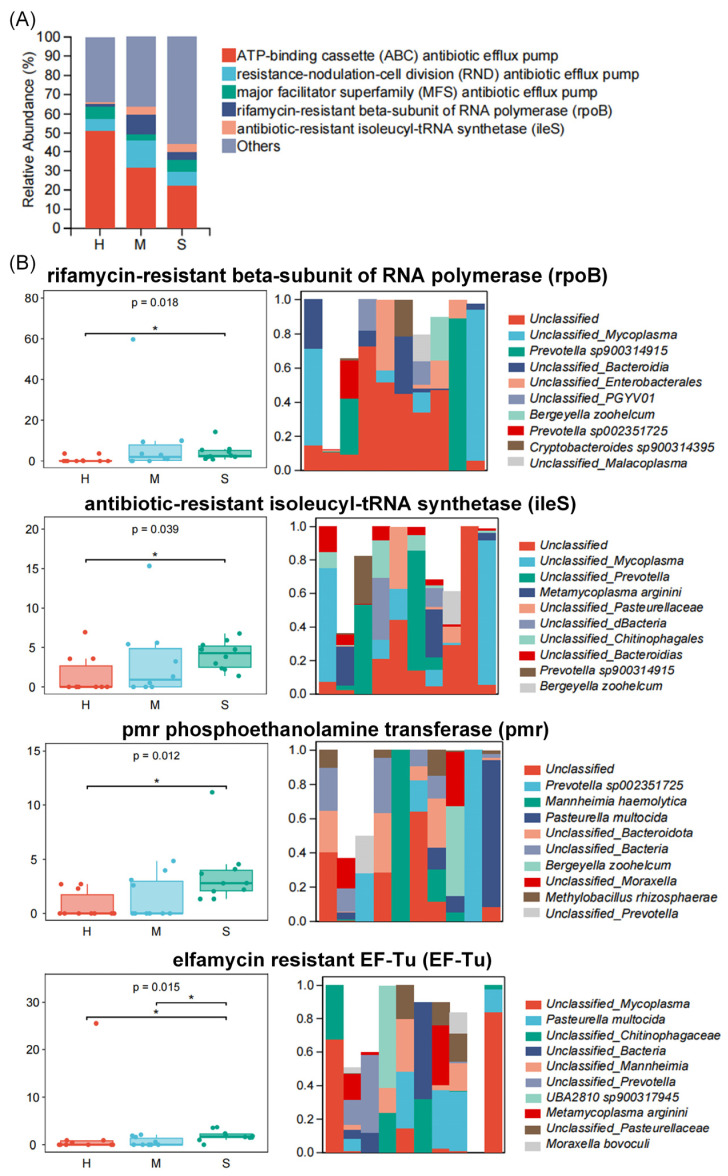
Putative antibiotic resistance genes (ARGs) in the lung microbiome across pneumonia severity groups. (**A**) Relative abundance of the top 5 ARG families. (**B**) Boxplots of differentially enriched putative ARGs, including rpoB, ileS, pmr, and EF-Tu, in the severe pneumonia group (* *p* < 0.05).

**Figure 10:**
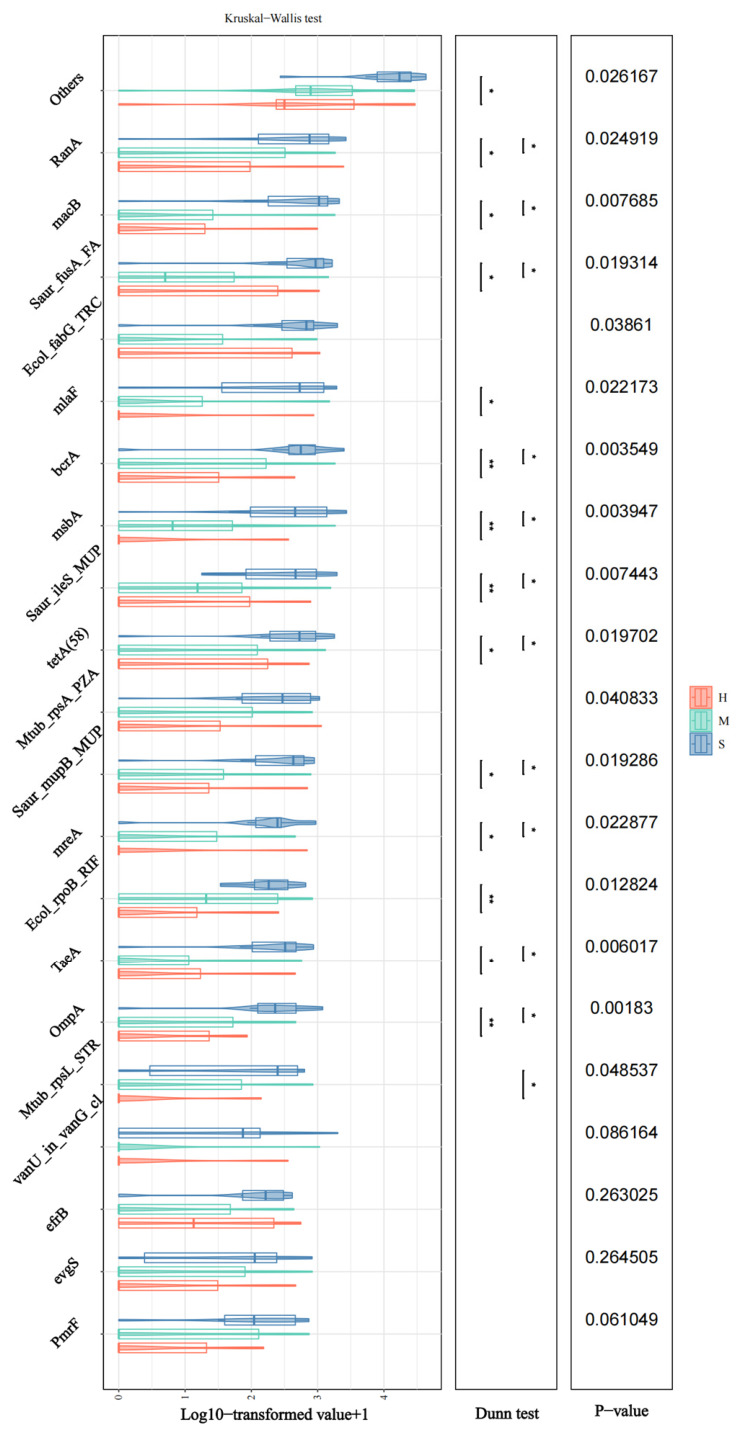
Differential abundance of specific putative antibiotic resistance genes across pneumonia severity groups. Only genes with significant differences are shown (* *p* < 0.05, ** *p* < 0.01).

Putative antibiotic resistance gene analysis showed significant enrichment of aminoglycoside, tetracycline, and polymyxin resistance genes in the severe pneumonia group. The dominant resistance mechanisms were ATP-binding cassette (ABC) transporters and resistance–nodulation–cell division (RND) efflux systems (Figure 9). Specific putative resistance genes, such as the rifamycin-resistant beta-subunit of RNA polymerase and antibiotic-resistant isoleucyl-tRNA synthetase, were also significantly enriched in the severe pneumonia group (Figure 10).

**Figure 11:**
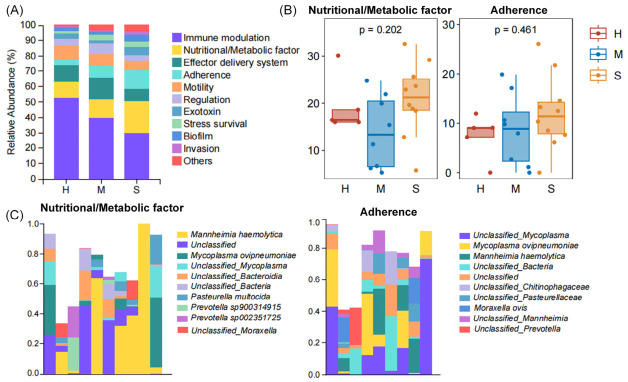
Virulence factor profiles of the lung microbiome across pneumonia severity groups. (**A**) Relative abundance of the top 5 virulence categories. (**B**) Boxplots of differentially enriched virulence categories. (**C**) Species contribution to virulence factor enrichment in the severe pneumonia group: Nutritional/Metabolic factor (left panel) and Adherence (right panel).

Virulence factor abundance analysis identified nutritional/metabolic factors and adherence as the primary virulence factors (Figure 11A). In the severe pneumonia group, these virulence functions were mainly associated with the genera *Moraxella*, *Mycoplasma*, *Pasteurella*, and *Acinetobacter*, with significant enrichment observed (*p* < 0.05) (Figure 11B,C). These findings suggest that the functional gene differences in the sheep lung microbiome are associated with pneumonia severity.

## 4. Discussion

Currently, although studies indicate that the lung microbiota significantly affects pneumonia onset and progression, the relationship between respiratory microbiome diversity and lung diseases remains contentious [14,15,16]. This study found that the lung microbiome richness and diversity in sheep with severe pneumonia were significantly higher than those in the healthy/mild lesion group. This finding aligns with research on the diversity of the pneumonia microbiome in broiler chickens, indicating that exposure to particulate matter leads to evident lung damage in broilers, resulting in significant alterations in lung microbiome structure and increased richness [22]. However, this conclusion contradicts findings in patients with community-acquired pneumonia, which suggest that higher lung microbiome diversity and richness contribute to the maintenance of pulmonary homeostasis [23]. Additionally, beta diversity analysis results indicate notable differences in the composition of the lung microbiomes between healthy/mild and severe pneumonia groups. These results suggest that the lung microbiota composition differs across lesion severity categories. It has been hypothesized that stressors may decrease host resistance, potentially leading to pathogen colonization and disruption of pulmonary microbial balance, thereby contributing to lung disease progression. However, further studies with true healthy controls are needed to test this hypothesis in the context of sheep pneumonia [24].

Subsequently, the analysis of the lung microbiome structure in sheep revealed that the microbiomes of both the healthy/mild lesion group and the pneumonia-affected groups were primarily composed of the phyla *Bacteroidetes*, *Firmicutes*, *Proteobacteria*, *Campylobacterota*, and *Actinobacteria*. Research indicates that *Bacteroidetes* is the most abundant group in the lung microbiome of sheep, with higher abundance observed in the severe pneumonia group. *Bacteroidetes*, as an important gut microbe, participates in various metabolic activities, such as carbohydrate fermentation and bile acid biotransformation; however, its specific functions within the lung microbiome remain unclear. Previous studies have identified an enrichment of *Bacteroides* in the bronchoalveolar lavage fluid of patients with sepsis and acute respiratory distress syndrome, significantly correlating with the inflammatory mediator TNF-α [25], suggesting a potential association with lung disease progression.

Moreover, the abundance of *Firmicutes* was higher in the healthy/mild lesion group than in the severe pneumonia group. *Firmicutes* contains beneficial genera such as *Lactobacillus*, which have been reported to be associated with the maintenance of pulmonary microbial balance and immune function [26,27]. At the genus level, a higher abundance and variety of respiratory-associated genera were found in the severe pneumonia group, including *Pasteurella*, *Mannheimia*, *Mycoplasma*, *Bibersteinia*, and *Moraxella*. This study confirmed that the degree of polymicrobial co-infection was significantly correlated with the severity of pulmonary lesions. This finding is consistent with our previous observation in goats [12], further supporting that mixed infection is associated with pneumonia progression in ruminants. The cross-sectional nature of this study means these findings represent associations rather than causal relationships.

Antibiotic resistance gene analysis identified the presence of various enriched resistance genes across all groups, primarily categorized into five major families, including ATP-binding cassette (ABC) antibiotic efflux pumps and resistance–nodulation–cell division antibiotic efflux pumps. These resistance mechanisms reduce the intracellular concentration of antibiotics or alter drug targets, diminishing the antibacterial effectiveness of these drugs. Compared to the healthy/mild lesion group, the severe pneumonia group exhibited significant differences in putative resistance genes, including aminoglycoside, tetracycline, polymyxin, and streptomycin resistance genes. The enrichment of these resistance genes may be associated with the prolonged and frequent use of multiple antibiotics in treating pneumonia-affected livestock. However, detailed information on antimicrobial treatment history and management practices was not available, as the animals were sourced from commercial farms. Additionally, animals with severe pneumonia typically have compromised immune function, which may be associated with increased susceptibility to colonization and resistance gene expression. These putative resistance genes are metagenomically predicted and require phenotypic confirmation. It should be noted that negative controls were not included during DNA extraction, PCR amplification, library preparation, or sequencing in this study, which represents a limitation for low-biomass samples and should be addressed in future studies. Future studies incorporating culture-based antimicrobial susceptibility testing are needed to confirm these findings.

In the analysis of virulence factors, it was found that nutrient/metabolic factors and adhesion were the main enriched virulence functions in the severe pneumonia group. Nutrient/metabolic factors play a crucial role in the virulence of pathogens, particularly in iron uptake. Iron is an essential nutrient for bacterial growth and metabolism, and iron deficiency can significantly affect bacterial survival and virulence [28]. Important virulence genes such as FbpABC and Shu assist bacteria in efficiently acquiring iron from the host environment, thereby supporting bacterial growth and enhancing virulence [29]. Bacterial adhesion to host cells represents a crucial early event during infection, enabling pathogens to resist host immune clearance and facilitating their establishment and multiplication on mucosal surfaces [30]. The virulence determinants, including capsule, EF-Tu, and the P97/P102 paralog family, enriched in the severe pneumonia group, are all associated with adhesion. Capsule enhances bacterial colonization, while EF-Tu helps bacteria evade host immunity and invade the host [31].

According to species contribution analysis, various bacterial species, including *Mannheimia*, *Mycoplasma*, *Pasteurella*, and *Moraxella*, were identified as the predominant contributors to the enrichment of nutrient/metabolic factors and adhesion-related virulence types. These results further highlight the intricate relationships between microbial communities and respiratory diseases, suggesting that future strategies for controlling sheep pneumonia should consider the impacts of microbial communities, antibiotic resistance, and virulence factors.

In summary, this study highlights compositional differences in microbial communities and their metabolic activities across sheep pneumonia severity categories, underscoring the associations between bacterial communities and pulmonary diseases. These findings provide new insights and suggest further research into the roles of specific microorganisms. It should be noted that, due to the limited number of completely lesion-free animals (*n* = 2), the healthy/mild lesion group included both animals with no visible lesions and those with mild lesions; thus, the comparisons reflect differences across lesion severity categories. Future studies with larger sample sizes, broader geographic coverage, and true healthy controls would further strengthen these findings.

## 5. Conclusions

This study employed 16S rRNA sequencing and metagenomic sequencing to thoroughly analyze the structural characteristics and functional genes of the lung microbiome in sheep. Our findings showed that the diversity and richness of the lung microbiome were higher in the severe pneumonia group. Genera such as *Pasteurella*, *Mannheimia*, *Mycoplasma*, *Bibersteinia*, and *Moraxella* were identified as enriched in severe cases. Additionally, the potential association between certain intestinal and oral microbiota and pneumonia may warrant further investigation. Regarding functional genes, carbohydrate and amino acid metabolism pathways were enriched in the severe pneumonia group. Putative antibiotic resistance genes were also abundant in this group, and nutritional/metabolic factors and adhesion were identified as the main virulence functions. Overall, this study provides a description of the functional characteristics of the lung microbiome in sheep with severe pneumonia and suggests the contribution of these genera to functional gene enrichment, providing a reference for future research on the prevention and treatment.

## Data Availability

The data presented in this study are available on request from the corresponding author due to ongoing analyses and further experiments.

## Supplementary Materials

The following supporting information can be downloaded at: https://www.mdpi.com/article/10.3390/vetsci13070679/s1. Figure S1. Electrophoresis detection figure of total DNA from selected samples. M: Marker; 49–95: DNA sample; CK: Negative. Figure S2. Dilution curves.

## Abbreviations

The following abbreviation is used in this manuscript:

ASVs	Amplicon Sequence Variants
