# Supplementary material for: Integrated 16S rRNA and Metagenomic Analysis of Pulmonary Microbiota in Sheep with Pneumonia
Source: Vet Sci. 2026 Jul 13;13(7):679. doi: 10.3390/vetsci13070679 (PMC13419273; doi:10.3390/vetsci13070679)
Supplement: Supplementary file 1 [file vetsci-13-00679-s001.zip › vetsci-4390594-supplementary.pdf]

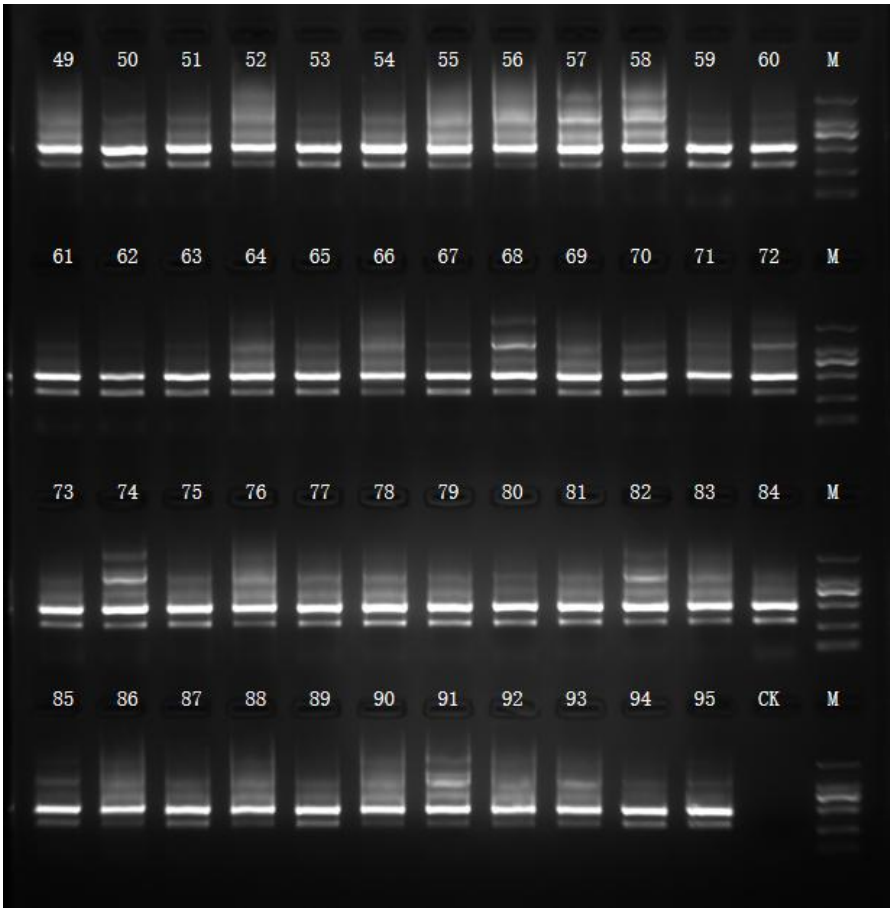

Figure S1. Electrophoresis detection figure of total DNA from selected samples
M: Marker; 49-95: DNA sample; CK: Negative

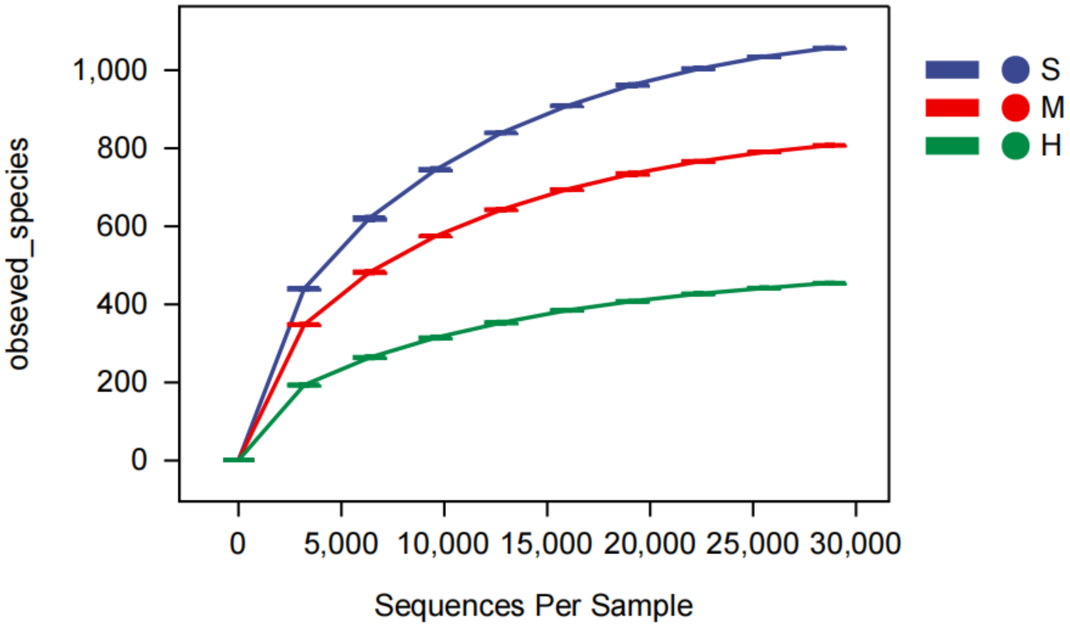

Figure S2. Dilution curves
